# Quality-controlled newborn hearing screening in Ghana: Outcomes and challenges from a pilot project

**DOI:** 10.4102/jcmsa.v4i1.396

**Published:** 2026-09-12

**Authors:** Estella Bilson-Amoah, Jemima Fynn, Emmanuel D. Kitcher, Latifa Seidu, Eric Manu

**Affiliations:** 1 Ear, Nose, and Throat Unit, Department of Surgery, Korle-bu Teaching Hospital, Accra, Ghana; 2 Audiology Centre, Faculty of Audiology, Speech and Language, Korle-bu Teaching Hospital, Accra, Ghana; 3 Department of Surgery, Faculty of Ear, Nose and Throat Surgery, University of Ghana Medical School, Accra, Ghana; 4 Department of Audiology, Faculty of Audiology, Speech and Language, University of Ghana, Accra, Ghana; 5 Department of Audiology, Faculty of Ear, Nose and Throat Surgery, Sunyani Teaching Hospital, Accra, Ghana

**Keywords:** Ghana, newborn hearing screening, neonatal hearing loss, UNHS, feasibility, public health

## Abstract

**Background:**

Neonatal hearing impairment affects 1–2 per 1000 live births in high-income countries, making early detection and habilitation critical before 6 months of age to ensure optimal language and communication development. Ghana currently lacks national legislation supporting universal newborn hearing screening (UNHS).

**Methods:**

This multicentre, hospital-based pilot study analysed newborn hearing screening data from four facilities between July 2023 and January 2025. Screening was conducted by trained staff using transient-evoked otoacoustic emissions and automated auditory brainstem response. Infants requiring follow-up were referred to the diagnostic centre at Korle-Bu Teaching Hospital. Results were documented, communicated to caregivers, and securely transferred to a centralised tracking system (pathTrack). Data analysis was performed using Microsoft Excel and SPSS version 20.0.

**Results:**

Of 29 075 live births, 11 107 (38.2%) were registered in the screening database, and 11 058 (99.6%) underwent screening at a median age of 13 days. Of these, 9007 (81.5%) passed the initial screening. Among 10 019 infants completing the process, three were diagnosed with permanent congenital hearing loss. Challenges included low coverage due to limited personnel, restricted diagnostic access, financial hardship, stigma, and barriers to follow-up. Caregivers demonstrated high acceptance of the screening process.

**Conclusion:**

This pilot demonstrates that newborn hearing screening in Ghana is feasible but constrained by systemic challenges. Strengthening infrastructure, expanding the trained workforce, ensuring sustainable funding, and increasing public awareness are essential to scaling up and sustaining a national UNHS programme.

**Contribution:**

This study provides the first quality-controlled evidence of newborn hearing screening feasibility in Ghana, demonstrating high parental acceptance while revealing systemic barriers such as limited personnel, restricted diagnostic access, financial hardship, and high loss to follow-up. By documenting outcomes and challenges across multiple hospitals, the research highlights critical pathways for scaling up UNHS in resource-limited settings. These insights align with JCMSA’s mission to strengthen clinical practice, public health systems, and medical education across Africa by providing context-specific evidence to guide policy integration, workforce development, and sustainable programme implementation.

## Introduction

Hearing impairment is a significant public health concern, with an estimated incidence of 6 per 1000 live births in resource-poor countries compared with 2–4 per 1000 in industrialised nations.^1^ Early diagnosis and intervention before 6 months of age resulted in improved communication and language outcomes.^2^ Since the 1990s, universal newborn hearing screening (UNHS) has been facilitated by automated methods such as automated auditory brainstem response (AABR) and transient-evoked otoacoustic emission (TEOAE).^3^ In the United States and many European countries, national or state-wide UNHS programmes are mandated.^3^ Updated guidelines have continued to refine these principles, emphasizing early detection and intervention.^4^ Across Africa, however, the situation is markedly different. Most countries lack mandated national or regional UNHS programmes, primarily due to systemic resource constraints, limited infrastructure, and insufficient trained personnel. Screening is often risk-based, targeting only infants with identifiable risk factors, which results in nearly half of affected newborns being missed. Studies from Kenya, Uganda, and Nigeria have demonstrated both the feasibility and challenges of implementing pilot UNHS programmes, highlighting issues such as high loss to follow-up, equipment shortages, and caregiver financial hardship.^5,6,7,8^ Compared with high-income countries where coverage rates exceed 95% and loss to follow-up is minimal, African programmes frequently report coverage below 50% and attrition rates exceeding 30% – 60%. These disparities underscore the urgent need for context-specific strategies to improve early detection and intervention for hearing loss across the continent. Ghana exemplifies this reality: despite being a regional leader in healthcare, it currently lacks a formal UNHS programme, relying on unsystematic risk-based screening that misses approximately 50% of affected infants.^9^ This challenge has been widely recognised, with calls for context-specific strategies to ensure feasibility in resource-limited settings.^10,11^ This pilot study was therefore designed to assess the feasibility of a quality-controlled UNHS programme in Ghana, aligned with international standards but responsive to local challenges.

Neonatal hearing impairment can result in long-term consequences, including delayed communication, emotional and cognitive difficulties, and challenges in future employment^1^. Prior to 1993, only high-risk infants in the United States were screened.^9^ Following National Institutes of Health (NIH) recommendations, all states in the United States implemented UNHS by 1999, with screening before discharge and intervention by 6 months of age.^12,13^ Subsequent position statements reinforced these recommendations, broadening programme standards and quality indicators.^4,14^

Most countries without UNHS are resource-constrained,^9^ facing challenges such as inadequate infrastructure, limited trained personnel, and insufficient funding. Across Africa, these systemic barriers have resulted in fragmented or pilot-level programmes rather than national mandates. Screening is often risk-based, targeting only infants with identifiable risk factors, which leaves nearly half of affected newborns undetected. Ghana exemplifies this reality: despite being a regional leader in healthcare, it currently lacks a formal UNHS programme. Screening is conducted on a risk-based basis, missing approximately 50% of infants with congenital hearing loss who present without obvious risk indicators.^9^ This gap means that many children are diagnosed late, often after critical windows for language and cognitive development have passed. The Ghanaian pilot study therefore provides crucial evidence of feasibility, while also highlighting the urgent need for policy integration, workforce expansion, and sustainable funding to bridge the disparity between international benchmarks and local realities.

While maternal and child health policies – such as the Child Health Record Book and immunisation programmes – provide potential platforms for integration, diagnostic services remain concentrated in tertiary centres, with limited trained personnel and funding. Without explicit policy endorsement and decentralised infrastructure, UNHS risks remaining peripheral to child health services despite its feasibility.

## Research methods and design

### Study design and setting

The study was conducted at four hospitals (Komfo Anokye Teaching Hospital [KATH], Korle-Bu Teaching Hospital [KBTH], 37 Military Hospital, Greater Accra Regional Hospital [GARH]) between July 2023 and January 2025.

#### Korle-Bu Teaching Hospital

- **Setting:** Urban, located in Accra, Ghana’s capital city.
- **Level of Care:** Tertiary referral centre and the largest teaching hospital in Ghana.
- **Capacity or Patient Volume:** ~2000 beds, serving tens of thousands annually, with referrals nationwide.
- **Role in Pilot:** Hosted the central tracking and diagnostic centre, with advanced audiological testing and Ear, Nose and Throat (ENT) services.

#### Komfo Anokye Teaching Hospital

- **Setting:** Urban, Kumasi (Ashanti Region), Ghana’s second-largest city.
- **Level of Care:** Tertiary referral hospital serving the middle belt of Ghana.
- **Capacity or Patient Volume:** ~1200 beds, serving millions across central Ghana.
- **Role in Pilot:** Provided screening services in a high-volume urban setting, with access to specialist staff but limited decentralised diagnostics.

#### Greater Accra Regional Hospital

- **Setting:** Urban, Accra.
- **Level of Care:** Secondary referral hospital, serving as the main regional facility for Greater Accra.
- **Capacity or Patient Volume:** ~450 beds, with thousands of deliveries annually.
- **Role in Pilot:** Illustrated screening feasibility in a busy regional hospital with limited specialist ENT support, highlighting the need for task-sharing with midwives and nurses.

#### 37 Military Hospital

- **Setting:** Urban, Accra.
- **Level of Care:** Secondary and tertiary facility, serving both military personnel and civilians.
- **Capacity or Patient Volume:** ~700 beds, moderate patient volume, with a mix of routine obstetric care and specialist services.
- **Role in Pilot:** Demonstrated feasibility in a mixed civilian-military hospital with structured governance but smaller newborn throughput compared with KBTH and KATH.

Primary screening and follow-up examinations were performed using TEOAE and/or AABR. A central tracking centre was established at KBTH.

### Study population

All newborns aged 0–3 months, with or without risk factors, were eligible. Exclusion criteria included infants older than 3 months, medically unstable Neonatal Intensive Care Unit (NICU) admissions, and lack of parental consent (see Table 1).

**TABLE 1:** Distribution of newborns registered and screened across the four participating health facilities in Ghana.

Facility	Registered and screened neonates
Korle-Bu Teaching Hospital (KBTH)	4058
Komfo Anokye Teaching Hospital (KATH)	2260
Greater Accra Regional Hospital (GARH)	3812
37 Military Hospital	928

**Total**	**11 058**

### Sampling and data collection

Parents received information and provided consent. Information about the screening procedure was provided to parents primarily through printed fliers and verbal explanations. Fliers were prepared in *English*, which is the official language of health communication in Ghana, but verbal explanations were often given in parents’ *home languages* (such as Akan or Twi, Ga, or Ewe) depending on the facility and staff capacity. This bilingual approach helped ensure comprehension across diverse linguistic backgrounds. Parental literacy varied: while many caregivers were literate and able to read the fliers, a significant proportion required verbal explanations due to limited literacy or preference for their home language. Consent was therefore obtained either in written form (for literate parents) or verbally (for those unable to read the fliers), ensuring inclusivity and ethical compliance.

Screening was generally performed within the first 20 days of life. Each infant was assigned a unique 12-digit ID. Risk factors were documented. Data were transmitted securely to a central server.

### Screening procedure

Thirty-three screeners (audiologists, otolaryngologists, midwives, paediatric nurses) were trained. A total of *33 healthcare professionals* were trained to conduct newborn hearing screening across the four pilot sites. The cadre of trained staff included *audiologists (20), otolaryngologists (3), midwives (10), and paediatric nurses (2)*, ensuring multidisciplinary participation. Despite this training, only 1–2 personnel per facility consistently performed the actual screening during the pilot, reflecting challenges in staff availability and competing clinical responsibilities (see Table 2). Screeners were trained and certified under the direct supervision of a screening and tracking specialist from *PATH MEDICAL GmbH, Germany*, who oversaw both the training sessions and competency assessments to ensure adherence to international standards.

**TABLE 2:** Comparison of neonates screened, number of personnel trained, and personnel consistently engaged in newborn hearing screening across the four participating health facilities in Ghana.

Facility	Neonates screened	Trained personnel	Actively screening (consistent)
KBTH	4058	6	2
KATH	2260	3	1
GARH	3812	4	1–2
37 Military Hospital	928	5	1

**Total**	**11 058**	**18 (of 33 trained)**	**5–6 consistently active**

Devices used included the SENTIERO Advanced^®^ and QSCREEN^®^. A two-stage screening protocol was implemented. At-risk infants underwent AABR alone. Tracking coordinators ensured follow-up compliance.

### Statistical analysis

Data were exported to Excel and analysed using descriptive statistics. Coverage, referral, and loss-to-follow-up rates were calculated. The data that were exported to Microsoft Excel and analysed using descriptive statistics came directly from the *screening outputs of the otoacoustic emissions (OAE) and auditory brainstem response (ABR) tests*:

- **From OAE and ABR devices:** Pass or fail results, device logs, and screening completion status were captured.
- **Linked variables:** Each infant’s anonymised ID was linked to outcome categories (PASS, FAIL, NO CONSENT, REJECTED, LTFU, CLOSED by TC, FU CLOSED, HL).
- **Barrier data:** Operational and caregiver-reported barriers were coded as categorical variables and linked to screening outcomes.
- **Analysis:** These datasets were exported into Excel, where descriptive statistics (frequencies, proportions, percentages) were calculated. Quality indicators (coverage, referral rate, loss to follow-up, diagnostic yield) were expressed as percentages using standard formulas.

### Ethical considerations

Ethical clearance to conduct this study was obtained from the Korle-Bu Teaching Hospital and Korle-Bu Teaching Hospital Institutional Review Board (No. 000211/2024). In addition, formal approval and administrative consent were granted by the heads of the participating institutions – Komfo Anokye Teaching Hospital (KATH), Greater Accra Regional Hospital (GARH), and 37 Military Hospital – prior to commencement of the pilot. This ensured that the study was conducted in accordance with institutional governance requirements and national ethical standards.

## Results

### Screening coverage

Screening coverage included (see Figure 1):

- Births recorded: 29 075
- Registered for screening: 11 107 (38.2%)
- Screened: 11 058 (99.6% of registered)
- Median age at screening: 13 days

**FIGURE 1:**
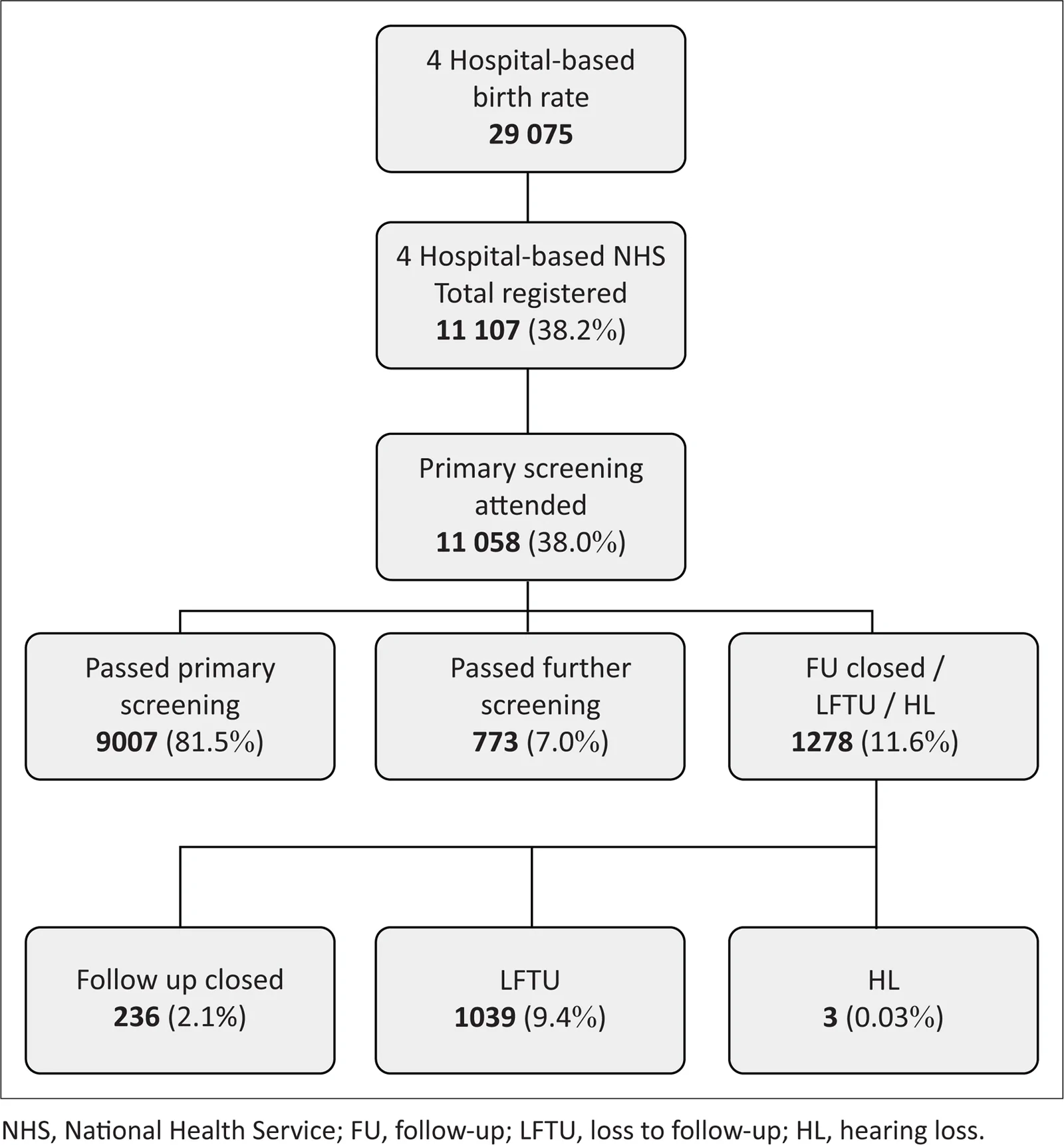
Flowchart of newborn hearing screening outcomes across four hospitals (July 2023–January 2025).

### Screening outcomes

The screen outcomes included:

- Pass rate (initial): 81.5% (9007)
- Referral rate: 18.5% (2051)
- Loss to follow-up: 50.7%
- Confirmed hearing loss: 3 infants (0.03%), all bilateral

Table 3 summarises the outcomes of all infants enrolled in the pilot newborn hearing screening programme across the four study sites. Each category reflects a distinct stage in the screening and tracking process.

**TABLE 3:** Outcomes of newborn hearing screening and follow-up tracking.

Category	PASS	FAILED	NO CONSENT	REJECTED	LTFU	CLOSED by TC	FU CLOSED	HL	Total
Total	773	55	9	11	742	222	236	3	2051

Note: PASS - Infants who passed the initial screening; FAILED - Infants who did not pass and required further testing; NO CONSENT - Cases where parental consent was not obtained; REJECTED - Invalid or incomplete tests; LTFU (Loss to Follow-Up) - Infants who did not return for confirmatory testing despite referral; CLOSED by TC - Cases closed administratively by the tracking centre; FU CLOSED - Follow-up completed and case closed; HL (Hearing Loss) - Infants confirmed with hearing loss after diagnostic assessment.

Total: Aggregate number of infants across all categories.

### Quality indicators

Quality indicators included:

- Referral rate: 18.5% (2051/11 058)
- Diagnostic yield: 0.03% (3 confirmed cases out of 10 019 completing screening)

From Table 1:

- Infants referred for rescreening = 2051
- Infants lost to follow-up (LTFU) = 742
- LTFU rate = 742 / 2051 × 100 = 36.2%

### Challenges identified

The main barriers to implementation included:

- Low coverage due to limited trained personnel.
- Restricted diagnostic access concentrated at a single centre.
- Financial hardship limits caregiver compliance.
- Fear of stigma associated with hearing loss.

Barriers to follow-up, including:

#### Missed Appointments

Many caregivers did not return for scheduled diagnostic assessments after initial referral. Reasons included competing family responsibilities, lack of awareness of the importance of follow-up, and logistical difficulties in arranging transport.

#### Loss to Follow-Up (LTFU)

A significant proportion of infants referred for diagnostic testing were never brought back to the facility. This was often due to poor tracking systems, limited reminder mechanisms, and absence of community-based follow-up structures.

#### Financial Hardship

Caregivers faced direct costs (transport, accommodation, meals) and indirect costs (time away from work). These financial burdens discouraged compliance, especially for families outside Accra who had to travel long distances to Korle-Bu Teaching Hospital.

#### Geographic and Infrastructure Barriers

Diagnostic services were centralised at KBTH, requiring families from Kumasi, GARH, and 37 Military Hospital to travel to Accra. Long travel distances and poor transport infrastructure compounded attrition.

#### Fear of Stigma

Some caregivers expressed reluctance to pursue follow-up due to cultural stigma associated with hearing loss. This was reported during telephone follow-ups and in-person interviews, where families feared labelling or discrimination.

#### Documentation and Systemic Gaps

Inconsistent record-keeping and limited integration of screening data into routine child health records led to missed opportunities for follow-up. Without robust electronic tracking, some infants were not flagged for recall. Despite these challenges, staff reported high acceptance among caregivers, with most parents willing to participate once informed of the screening benefits.

## Discussion

Coverage in this study (38.2%) fell short of the ≥ 95% international benchmark,^3^ largely due to limited trained personnel. Task-sharing with nurses and midwives, as implemented elsewhere,^15^ could improve coverage. Equipment failures disrupted screening, underscoring the need for redundancy.

Parental acceptance was high (99.6%), consistent with Kenya^5^ and Uganda.^6^ However, loss to follow-up was 50.7%, similar to Uganda (65.7%)^11^ and Nigeria (51.6%),^8^ but much higher than Germany (1.0%)^16^ and France (7.7%).^17^ A controlled trial in the United Kingdom which demonstrated the effectiveness of universal newborn screening.^18^ Predictors of attrition in developing countries have been studied, highlighting socioeconomic and systemic barriers.^19^

The primary pass rate (81.5%) exceeded South Africa (57%),^7^ but the referral rate (18.6%) was higher than the recommended ≤ 4%.^3^ High ambient noise contributed, with ward levels exceeding the World Health Organization (WHO) guidelines.^20,21^ Vernix caseosa and middle ear fluid also impacted screening.^22,23,24^

Three infants (0.03%) – equivalent to *0.3 per 1000 live births* – were diagnosed with bilateral congenital hearing loss, all with identifiable risk factors. This prevalence is markedly lower than Côte d’Ivoire (6/1000), Nigeria (28/1000), and India (5–6/1000), but is likely underestimated due to low coverage and high loss to follow-up.^25^

Key barriers to implementation extended beyond operational challenges to systemic weaknesses. Low coverage reflected workforce shortages and limited integration into routine care. Restricted diagnostic access at KBTH created inequity for families outside Accra, while financial hardship compounded attrition. Stigma discouraged caregivers from pursuing follow-up, reflecting cultural barriers to acceptance. Poor tracking, evidenced by 742 infants lost to follow-up and 222 prematurely closed cases, highlighted weaknesses in data management and recall systems. Together, these barriers underscore the need for workforce expansion, decentralisation of diagnostics, financial support mechanisms, community education, and strengthened tracking systems to ensure programme sustainability.

### Limitations of the study

This pilot study has several limitations that should be acknowledged. Firstly, the screening was conducted only in four urban, high-volume hospitals, which may not reflect the realities of rural or primary-level facilities where infrastructure and personnel are more limited. Secondly, diagnostic confirmation was restricted to Korle-Bu Teaching Hospital, creating geographic and financial barriers that contributed to high loss-to-follow-up rates and limited generalisability of diagnostic outcomes. Thirdly, the reliance on caregiver self-report during follow-up introduced potential recall and reporting bias, particularly regarding stigma and financial hardship. Fourthly, documentation inconsistencies and occasional premature closure of cases in the tracking system may have led to underestimation or misclassification of certain barriers. Finally, as a pilot, the study was not powered to estimate national prevalence of congenital hearing loss, and the small number of confirmed cases limits conclusions about disease burden.

### Recommendations

The study makes the following recommendations:

- Institutional Integration: Incorporate screening into the Maternal and Child Health Handbook and 6-week immunisation visits.
- Infrastructure Expansion: Establish diagnostic centres near screening sites; ensure backup equipment.
- Workforce Development: Train nurses, midwives, and community health workers; provide continuous education.
- Awareness and Education: Integrate hearing loss modules into curricula; launch public campaigns. To ensure sustainability, newborn hearing screening should be integrated into existing health training programmes. Audiology and ENT curricula should focus on diagnostic protocols, while nursing and midwifery training should include routine screening skills. Community health workers should be taught awareness and referral pathways to support follow-up. Introducing sensory health topics in secondary school health science tracks can build early awareness and a pipeline of future professionals. Training audiologists, ENT specialists, midwives, paediatric nurses, community health workers, and health information officers through this integrated approach will expand the workforce and improve coverage.
- Tracking and Follow-Up: Implement digital tracking systems; use SMS or community reminders. The high proportion of infants lost to follow-up (36.2%) and the premature closure of 222 cases highlight weaknesses in tracking systems. Addressing these gaps requires integrating screening data into routine child health records, implementing automated recall alerts, and training health information officers in accurate case management. Involving community health workers in follow-up could further reduce attrition by providing localised support.
- Policy and Funding: Advocate for national UNHS endorsement; secure sustainable funding aligned with WHO guidelines. International consensus statements continue to stress the importance of embedding UNHS within broader child health frameworks.^4,25^

## Conclusion

This pilot demonstrates that newborn hearing screening in Ghana is feasible but constrained by systemic challenges. High parental acceptance provides a strong foundation for scale-up.
